# A *SUMO4* initiator codon variant in amyotrophic lateral sclerosis reduces *SUMO4* expression and alters stress granule dynamics

**DOI:** 10.1007/s00415-022-11126-7

**Published:** 2022-05-03

**Authors:** Alma Osmanovic, Alisa Förster, Maylin Widjaja, Bernd Auber, Anibh M. Das, Anne Christians, Frank Brand, Susanne Petri, Ruthild G. Weber

**Affiliations:** 1 Department of Human Genetics, Hannover Medical School, Carl-Neuberg-Straße 1, 30625 Hannover, Germany; 2 Department of Neurology, Hannover Medical School, Carl-Neuberg-Straße 1, 30625 Hannover, Germany; 3 Department of Pediatric Kidney, Liver and Metabolic Diseases, Hannover Medical School, Carl-Neuberg-Straße 1, 30625 Hannover, Germany

**Keywords:** Amyotrophic lateral sclerosis, Genetic risk, Head trauma, Cancer, SUMOylation, Stress granules

## Abstract

**Background:**

Recent evidence points toward a role of the small ubiquitin-like modifier (SUMO) system, including SUMO4, in protecting from stress insults and neurodegeneration, such as the progressive motor neuron disease amyotrophic lateral sclerosis (ALS), e.g., by regulating stress granule (SG) dynamics. Here, we investigated whether *SUMO4* variants play a role in ALS pathogenesis.

**Methods:**

Whole-exome or targeted *SUMO4* sequencing was done in 222 unrelated European ALS patients. The consequences of the identified initiator codon variant were analyzed at the mRNA, protein and cellular level. *SUMO4* expression was quantified in human tissues. All patients were subjected to clinical, electrophysiological, and neuroradiological characterization.

**Results:**

A rare heterozygous *SUMO4* variant, i.e., *SUMO4*:c.2T>C p.Met1?, was detected in four of 222 (1.8%) ALS patients, significantly more frequently than in two control cohorts (0.3% each). *SUMO4* mRNA and protein expression was diminished in whole blood or fibroblasts of a *SUMO4* variant carrier versus controls. Pertinent stress factors, i.e., head trauma or cancer (treated by radiochemotherapy), were significantly more frequent in *SUMO4* variant carrier versus non-carrier ALS patients. The mean number of SGs per cell was significantly higher in fibroblasts of a *SUMO4* variant carrier compared to controls at baseline, upon oxidative stress, and after recovery, and SUMOylation of ALS-associated valosin-containing protein by SUMO4 was decreased. *SUMO4* mRNA expression was highest in brain of all human tissues analyzed.

**Conclusions:**

Our results are consistent with *SUMO4* haploinsufficiency as a contributor to ALS pathogenesis impacting SG dynamics and possibly acting in conjunction with environmental oxidative stress-related factors.

## Introduction

Amyotrophic lateral sclerosis (ALS) is a progressive fatal neurodegenerative disease characterized by the loss of motor neurons. Mutations in ALS-associated genes, such as *FUS* (FUS RNA binding protein) and *VCP* (valosin-containing protein), as well as environmental stress and lifestyle factors are involved in ALS pathogenesis. In response to various stress conditions, stress granules (SGs), cytosolic membrane-less organelles consisting of untranslated mRNAs and RNA binding proteins (RBPs), form. Evidence is emerging that pathological aggregation of RBPs and persistent SGs play a role in neurodegeneration [1], and represent promising therapeutic targets [2].

In a mouse model recently reported by Zhang et al*.*, an ALS-associated *FUS* mutation was linked to SG misprocessing and motor performance decline aggravated by stress [3]. *FUS* encodes a nuclear RBP that, during the SG cycle, translocates to the cytoplasm where SGs nucleate, mature, and disperse [1]. Among other things, Zhang et al*.* reported that cultured motor neurons from *FUS* knock-in mice were prone to form SGs in response to oxidative stress, and showed decreased disassembly of SGs after recovery from stress challenge. These data corroborate and extend earlier findings in HEK293 cells and the zebrafish spinal cord that expression of a *FUS* nonsense mutation, known to cause ALS, resulted in cytoplasmic accumulation of mutant FUS, and assembly into SGs upon oxidative stress [4]. Furthermore, mutations in *VCP* and other ALS-associated genes have been shown to affect SGs under stress conditions [5, 6], suggesting that yet other SG-related proteins may be involved in ALS pathogenesis.

The distribution of VCP to SGs required for SG clearance [7] is facilitated by SUMOylation of VCP [5]. SUMOylation, a ubiquitination-like post-translational modification, involves the reversible covalent conjugation of a small ubiquitin-like modifier (SUMO) to lysine residues of a multitude of cellular proteins [8]. Recent evidence points toward a role of the SUMO system in protecting from ALS pathology, e.g., by regulating SG dynamics [5]. For instance, SG disassembly-engaged proteins include SUMO ligases that are recruited during normal clearance and dysregulated in ALS-like conditions [9]. Interestingly, SUMO E3 ligase activity has been described for FUS [10]. To our knowledge, no ALS-associated mutations have been reported in genes encoding SUMO isoforms 1–4. Here, we provide evidence that not only a *FUS* mutation, as described in the ALS mouse model by Zhang et al. [3], but also a specific variant in the *SUMO4* gene detected in ALS patients affects SG dynamics as an underlying pathomechanism of ALS.

## Patients and methods

### Subjects and their clinical characterization

The study was approved by the Ethics Board of Hannover Medical School (ID # 6269). Written informed consent was obtained from all individuals. At the ALS/motor neuron disease clinic of the Department of Neurology, Hannover Medical School, Hannover, Germany, 222 central European ALS patients (11 with familial ALS and 211 with sporadic ALS), and in some cases, their parents or siblings were recruited to the study. The median age at onset of patients was 61 years, and the male to female ratio was 1.4:1. Regarding the site of symptom onset, the proportions of subjects with initial bulbar and spinal symptoms were 23.4 and 76.6%, respectively. Patients were subdivided into eight clinical subtypes (upper motor neuron dominant ALS, bulbar phenotype, flail arm syndrome, flail leg syndrome, respiratory phenotype, progressive muscular atrophy, lower motor neuron dominant ALS, and classic (Charcot) ALS) as described previously [11]. Disease status was evaluated using the revised ALS functional rating scale (ALSFRS-R). At first presentation, the progression rate was calculated using the following formula: 48–total ALSFRS-R/symptom duration in months [12]. Of the proposed environmental stress and lifestyle factors increasing the risk of ALS [13], smoking habits, occupation, chemical exposure, and physical exercise were not assessed routinely, whereas history of head trauma and tumors with associated treatments, such as chemotherapy or radiation, could be retrieved from patients’ past medical records. Detailed information on the severity of traumatic head injury or the specific radiotherapy/chemotherapeutic regimens used was not available.

### Whole-exome/targeted sequencing and data analysis

DNA was extracted from whole blood samples using the QIAamp DNA Blood Maxi Kit (Qiagen, Hilden, Germany). Whole-exome sequencing (WES) was performed on leukocyte DNA of 24 ALS patients and 1787 in-house individuals. Target enrichment was done using the Agilent SureSelect Human All Exon v4 Target Enrichment System (Agilent Technologies, Inc., Santa Clara, CA, U.S.A.) or the xGen® Exome Research Panel (Integrated DNA Technologies, Inc., Coralville, U.S.A.). All samples were sequenced to a mean target coverage of >50× on an Illumina HiSeq 2000 or an Illumina NextSeq 500 using the NextSeq 500/550 High Output v2 kit (all Illumina, San Diego, CA, U.S.A.). Data were processed and aligned to the GRCh37/hg19 reference human genome build using the Biomedical Genomics Workbench (version 5.0; Qiagen) or megSAP, version 0.1-710-g52d2b0c (https://github.com/imgag/megSAP). Variant prioritization and visualization were performed using Ingenuity Variant Analysis (Qiagen), GSvar version 2018_04 (https://github.com/imgag/ngs-bits), IGV version 2.4.14 (https://software.broadinstitute.org/software/igv/), and Alamut® visual version 2.11 (Interactive Biosoftware, Rouen, France). Targeted sequencing of the coding and flanking intronic sequences of the *SUMO4* gene (NM_001002255) by a conventional chain termination protocol was used to verify *SUMO4* variants identified by WES, and to do mutational analysis of 198 additional ALS patients. Minor allele frequencies (MAF) of genetic variants were extracted from the Genome Aggregation Database (gnomAD) Browser v2.1.1 (https://gnomad.broadinstitute.org). Variant pathogenicity was predicted using SIFT (http://sift.jcvi.org/), PolyPhen-2 (http://genetics.bwh.harvard.edu/pph2/), and MutationTaster (http://www.mutationtaster.org).

### Quantitative *SUMO4* mRNA expression analysis in ALS patients and human tissues

RNA was isolated from whole blood of three ALS patients, i.e., *SUMO4* variant carrier TALS004-01 and two non-carriers, using the RNeasy Mini Kit (Qiagen). To eliminate DNA contamination, RNA samples were treated with RNase-free DNase I (Qiagen). cDNA synthesis was performed using the SuperScript III First-Strand Synthesis System (Thermo Fisher Scientific, Waltham, MA, U.S.A.). For quantitative *SUMO4* mRNA expression analysis, the TaqMan Universal PCR Master Mix and a TaqMan gene expression assay for *SUMO4* (Hs01940570_g1; Thermo Fisher Scientific) were used to analyze cDNA of ALS patients and Human Multiple Tissue cDNA (MTC) Panel I (#636742; Clontech-Takara Bio Europe, Saint Germain-en-Laye, France) in three and two independent experiments, respectively. Target gene expression levels were normalized to expression levels of *B2M* (Hs00187842_m1; Thermo Fisher Scientific), and comparative C_t_ quantification was applied.

### Cell culture of fibroblasts of patient TALS004-01 and controls

Skin biopsy-derived fibroblasts of patient TALS004-01 and of two age- and sex-matched non-ALS controls were cultured in Dulbecco’s Modified Eagle Medium (DMEM, Merck, Darmstadt, Germany) supplemented with 10% fetal bovine serum, 2 mM l-glutamine, and 1% penicillin/streptomycin (all Thermo Fisher Scientific).

### Targeted *SUMO4* sequencing on fibroblast DNA of patient TALS004-01 and controls

DNA was isolated from fibroblasts of patient TALS004-01 and the non-ALS controls using the innuPREP DNA Mini Kit (Analytik Jena, Jena, Germany). The coding sequence and parts of the flanking untranslated regions of *SUMO4* were amplified and sequenced using a conventional chain termination protocol. Sequence data were analyzed using the SeqPilot software v4.3.1 (JSI Medical Systems GmbH, Ettenheim, Germany).

### Western blot analysis of fibroblast lysates of patient TALS004-01 and controls

Fibroblasts were homogenized in lysis buffer (20 mM Tris–HCl, pH 8.0, 50 mM sodium fluoride, 1 mM sodium orthovanadate, 1% Nonidet P40, supplemented with protease inhibitors (Roche Diagnostics, Mannheim, Germany)), and protein concentration was determined using the Pierce BCA Protein Assay Kit (Thermo Fisher Scientific). Cell lysates were adjusted to equal protein concentrations, mixed with 4 × Laemmli buffer (250 mM Tris–HCl, pH 6.8, 40% glycerol, 8% sodium dodecyl sulfate (SDS), 20% 2-mercaptoethanol, 4 mM ethylenediaminetetraacetic acid (EDTA), 0.04% bromophenol blue (w/v)), and used in equal amounts for SDS–polyacrylamide gel electrophoresis and semidry electroblotting. After incubating polyvinylidene difluoride or nitrocellulose membranes (GE Healthcare, Chicago, IL, U.S.A.) in 5% fat-free milk powder dissolved in phosphate-buffered saline (PBS) with 0.05% Tween 20 (PBST) to block unspecific binding, the following primary antibodies were diluted in 5% bovine serum albumin (BSA) in PBST and incubated at 4 °C overnight for immunodetection: anti-GAPDH (#MAB374, Merck; dilution 1:3000), anti-pan-SUMO detecting SUMO1-4 (#A-714, R&D Systems, Minneapolis, MN, U.S.A.; dilution 1:1000), anti-SUMO4 [EPR7163], lot: GR155829-1 (#ab126606, Abcam, Cambridge, U.K.; dilution 1:1000), anti-VCP (#MA3-004; Thermo Fisher Scientific; dilution 1:1000). All membranes were exposed to the corresponding horseradish peroxidase-conjugated secondary antibody (Thermo Fisher Scientific) diluted at 1:3000 in 5% fat-free milk powder dissolved in PBST, and developed using the Pierce SuperSignal West Dura detection kit (Thermo Fisher Scientific). Images were acquired using the Fusion-FX7 gel documentation system (Vilber, Collégien, France). Protein bands were quantified using the ImageJ software (https://imagej.nih.gov/ij/). SUMO4, pan-SUMO and GAPDH protein levels were calculated from three independent experiments.

### Immunofluorescence of fibroblasts of patient TALS004-01 and controls to visualize stress granules

Fibroblasts (5.0 × 10^4^ cells) were seeded on fibronectin-coated glass coverslips. After 24 h, cells were treated with 0.5 mM sodium arsenite (NaAsO_2_) solution (Sigma-Aldrich, St. Louis, MO, U.S.A.) for 45 min at 37 °C (oxidative stress). After removal of NaAsO_2_, cells were incubated in DMEM with supplements for 60 min at 37 °C (recovery). Cells were fixed with 4% paraformaldehyde for 15 min at 4 °C, permeabilized with 0.25% Triton X-100 in PBS for 25 min at room temperature (RT), and incubated with 0.1% Triton X-100, 6% BSA in PBS for 1 h at RT to block unspecific binding. The primary anti-TIAR rabbit antibody (D32D3, #8509, Cell Signaling Technology, Danvers, MA, U.S.A.) was diluted at 1:1600 in 0.1% Triton X-100, 1% BSA in PBS and incubated overnight at 4 °C. After washing three times in PBS, the Alexa Fluor 488-conjugated anti-rabbit secondary antibody (Thermo Fisher Scientific; dilution 1:500) was incubated for 1 h at RT. After counterstaining with 4′,6-diamidino-2-phenylindole (DAPI), cells were mounted in Mowiol mounting medium. Images were captured using an epifluorescence microscope (Leica DM RXA2) equipped with a cooled charge-coupled device camera (SenSys, Photometrics, Tucson, AZ, U.S.A.). Quantification of TIAR-positive stress granules was performed using Fiji software [14]. Stress granules were arbitrarily defined as cytoplasmic foci of ≥ 0.5 µm in diameter staining positive for TIAR. At least 30 cells were analyzed per condition and individual in each of three independent experiments.

### Enrichment of VCP from fibroblast lysates of patient TALS004-01 and controls by immunoprecipitation

Fibroblasts were washed with ice-cold PBS and lysed for 30 min on ice in 1 ml of immunoprecipitation (IP) lysis buffer (20 mM Tris–HCl, pH 8.0, 50 mM sodium fluoride, 1 mM sodium orthovanadate, 1% Nonidet P40, supplemented with protease and phosphatase inhibitors; Roche Diagnostics). Cell lysates were incubated with 1 µg anti-VCP antibody (#MA3-004; Thermo Fisher Scientific) by tumbling over night at 4 °C. Protein G Sepharose beads (GE Healthcare) were equilibrated in IP lysis buffer and incubated with the cell lysates for 4 h at 4 °C. After six washing steps with IP lysis buffer, bound protein was eluted from the beads using 1 × Laemmli buffer (62.5 mM Tris–HCl, pH 6.8, 10% glycerol, 2% SDS, 5% 2-mercaptoethanol, 1 mM EDTA, 0.01% bromophenol blue). VCP and SUMO4-VCP were detected by Western blot analysis using anti-VCP and anti-SUMO4 antibodies, as described above. Protein bands were quantified using the ImageJ software (https://imagej.nih.gov/ij/). Levels of SUMO4-VCP and VCP immunoprecipitates were calculated from two independent experiments.

### Statistics

Statistical comparisons were made using two-tailed Fisher’s exact test, Mann–Whitney *U* Test or Chi-square test, as appropriate and indicated in table legends. Statistical significance of experiments was calculated using two-tailed Student’s *t* test or one-way ANOVA as indicated in the figure legend. The analysis was carried out using MATLAB (R2017a; The MathWorks, Inc., Natick, MA, U.S.A.), PRISM software (v.5; GraphPad software, San Diego, CA, U.S.A.) or SPSS (IBM® Statistical Software Package of Social Science, Chicago, IL, U.S.A.) version 26, *p*-values of ≤ 0.05 (*), ≤ 0.01 (**), and ≤ 0.001 (***) were considered statistically significant.

## Results

**Fig. 1:**
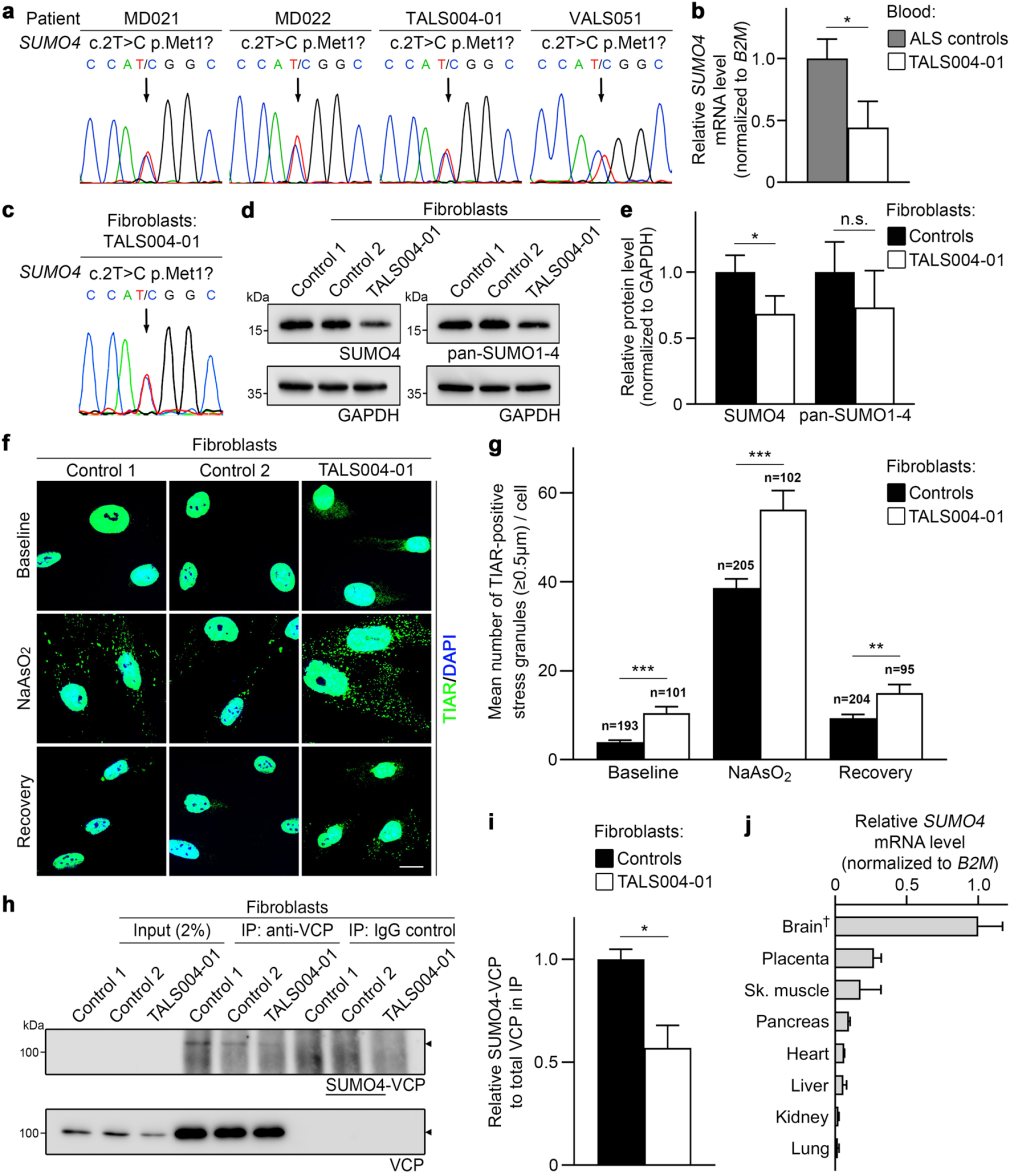
A rare heterozygous *SUMO4*:c.2T>C initiator codon variant detected in 1.8% of ALS patients leads to reduced *SUMO4* expression, altered stress granule dynamics, and reduced SUMOylation of VCP. **a** Electropherograms demonstrating the rare heterozygous *SUMO4*:c.2T>C p.Met1? variant in leukocyte DNA of four sporadic ALS patients. **b** *SUMO4* mRNA expression in whole blood of *SUMO4*:c.2T>C variant carrier TALS004-01 and two ALS control patients without the *SUMO4*:c.2T>C variant was quantified by real-time RT-PCR and normalized to *B2M* mRNA. *SUMO4* mRNA levels were significantly reduced in whole blood of patient TALS004-01 compared to ALS controls (mean ± SD of triplicate samples from three independent experiments). **c**–**e** Detection (**d**) and densitometric quantification (**e**) of unconjugated SUMO4 protein and all unconjugated SUMO isoforms 1–4 by Western blot analysis of lysates of fibroblasts of patient TALS004-01 harboring the *SUMO4*:c.2T>C variant (**c**) and of two sex- and age-matched non-ALS controls without rare *SUMO4* variants using anti-SUMO4 and anti-pan-SUMO (detecting SUMO isoforms 1–4) antibodies, showing that SUMO4 protein levels were significantly reduced and levels of all SUMO isoforms were reduced in fibroblasts of patient TALS004-01 compared to controls (mean ± SD from three independent experiments). **f**, **g** Detection (**f**) of stress granules (SGs) in fibroblasts of patient TALS004-01 and two non-ALS controls that were untreated (baseline), treated for 45 min with 0.5 mM sodium arsenite (NaAsO_2_) or had recovered for 60 min from NaAsO_2_ treatment (recovery) by immunostaining of the SG marker protein nucleolysin TIAR; nuclei were counterstained with DAPI; scale bar 10 µm. Quantification (**g**) of SGs from (**f**), showing significantly higher numbers of SGs in fibroblasts of patient TALS004-01 compared to fibroblasts of non-ALS controls at baseline, upon NaAsO_2_, and after recovery (mean ± SEM of ≥ 95 cells per individual and condition from three independent experiments with at least 30 cells per individual, condition and experiment). **h**, **i** Immunoprecipitation (IP) of VCP, and detection (**h**) and densitometric quantification (**i**) of SUMO4-conjugated VCP by Western blot analysis of fibroblast lysates of patient TALS004-01 and non-ALS controls using an anti-SUMO4 antibody, showing that VCP is SUMOylated by SUMO4 and that SUMOylation of VCP by SUMO4 is significantly reduced in fibroblasts of patient TALS004-01 compared to controls (mean ± SD from two independent experiments). **j** *SUMO4* mRNA expression in a human adult multiple tissue cDNA panel was quantified by real-time PCR, normalized to *B2M* mRNA, and displayed relative to mRNA levels in brain (^†^). *SUMO4* mRNA levels were highest in brain followed by placenta and skeletal (sk.) muscle (mean ± SD of duplicate samples from two independent experiments). *p < 0.05; **p ≤ 0.01; ***p ≤ 0.001; *n.s.* not significant; according to two-tailed Student’s *t* test (**b**, **e**, **i**) or one-way ANOVA (**g**)

With the aim to identify new ALS-associated genes and to investigate whether rare *SUMO4* variants play a role in ALS pathogenesis, we analyzed leukocyte DNA of 222 unrelated central European ALS patients using whole-exome or targeted *SUMO4* sequencing. We detected an identical initiator codon variant in the *SUMO4* gene, NM_001002255(*SUMO4*):c.2T>C p.Met1?, in four of the 222 ALS patients, verified to be heterozygous by Sanger sequencing (Table 1; Fig. 1a). The four ALS patients carrying the *SUMO4*:c.2T>C variant were of German origin, but from different German regions/federal states, and, thus, do not belong to a specific subpopulation. The *SUMO4*:c.2T>C variant (with a MAF < 0.2%, Table 1) as well as all rare (MAF < 0.2%) *SUMO4* variants predicted to be deleterious by SIFT and/or PolyPhen-2 (according to the Genome Aggregation Database, v2.1.1) taken together were significantly more frequent in central European ALS patients (4/222 (1.8%), both comparisons) compared to in-house individuals mostly of German origin (5/1786 (0.3%), *p* = 0.0117 and 7/1786 (0.4%), *p* = 0.0256, two-tailed Fisher’s exact test) and non-Finnish European controls from the Genome Aggregation Database, v2.1.1, controls (74/24,101 (0.3%), *p* < 0.0001 and 90/20,432 (0.4%), *p* = 0.0027, Chi-square test). None of the four *SUMO4*:c.2T>C variant carriers harbored a hexanucleotide repeat expansion or pathogenic variant in the ALS-associated genes *C9orf72* (not analyzed in VALS051), *FUS, SOD1*, *TARDBP*, or *VCP*, or had a family history of ALS. None of the 24 ALS patients analyzed by WES carried a rare (MAF < 0.2%) variant in the *SUMO1*, *SUMO2* or *SUMO3* gene. The *SUMO4*:c.2T>C variant was also identified in the healthy father of variant carrier TALS004-01, the only parent available for genetic testing, suggesting either reduced penetrance of the variant, later onset of ALS in the father, or combined genetic and environmental causes of ALS in the patient. The latter assumption is corroborated by the fact that exposure to certain potentially pertinent oxidative stress-related factors, i.e., head trauma or tumor with and without treatment by radiation or chemotherapy, were significantly more frequent in patients carrying the *SUMO4*:c.2T>C variant compared to non-carriers in our ALS cohort (*p* = 0.012; Tables 2, 3). Patient TALS004-01 presented with a history of head trauma at 6 years of age (Table 3), whereas no stress factor has been reported in his father. Other characteristics of *SUMO4*:c.2T>C variant carriers versus non-carriers were not significantly different in our ALS cohort (Table 2).

**Table 1:** Characteristics of a *SUMO4* initiator codon variant identified in 4 of 222 (1.8%) central European patients with ALS

Patients	Chromosomal position GRCh37/hg19	Nucleotide change	Amino acid change	dbSNP	MAF^a^	Pathogenicity prediction		
						MutationTaster	SIFT	PolyPhen-2
MD021 MD022 TALS004-01 VALS051	6:149721529	c.2T>C	p.Met1?	rs17875292	0.001535	Disease causing	Deleterious	Possibly damaging

^*a*^*MAF*, minor allele frequency according to the Genome Aggregation Database Browser (Beta) gnomAD v2.1.1 (controls), European (non-Finnish), accessed March 2021

Reference sequence used: NM_001002255

**Table 2:** Comparison of clinical characteristics in our cohort of 222 ALS patients with or without the *SUMO4*:c.2T>C variant

Characteristics	*SUMO4*:c.2T>C carriers (*n* = 4) Number (%) or mean (range)	*SUMO4*:c.2T>C non-carriers (*n* = 218) Number (%) or mean (range)	*p* value*
Inheritance			
Sporadic	4 (100)	207 (95.0)	1.0
Sex			
Male	3 (75)	126 (57.8)	0.641
Age at onset, years	61 (29–73)	59.15 (25–84)	0.394
Duration, years	5.38 (1.75–9.58)^a^	3.86 (0.42–18.0)^b^	0.217
Site of onset			
Bulbar	1 (25)	51 (23.4)	1.0
Spinal	3 (75)	167 (76.6)	1.0
ALS-subtype			
Classic (Charcot) ALS	2 (50)	148 (67.9)	0.597
Upper motor neuron	1 (25)	7 (3.2)	0.137
Lower motor neuron	0 (0)	14 (6.4)	1.0
Flail arm	0 (0)	22 (10.1)	1.0
Flail leg	0 (0)	2 (0.92)	1.0
Progressive muscular atrophy	0 (0)	4 (1.8)	1.0
Bulbar	0 (0)	19 (8.7)	1.0
Respiratory	1 (25)	2 (0.92)	0.053
ALSFRS-R progression rate	0.38 (0.33–0.45)^c^	0.66 (0.04–3.6)^d^	0.552
Environmental stress factor^e^	3 (75.0)^f^	31 (14.2)	**0.012**

*ALS* amyotrophic lateral sclerosis, *ALSFRS-R* amyotrophic lateral sclerosis functional rating scale revised

^a^Until last follow up (TALS004-01) or death (MD021, MD022, VALS051)

^b^Until last follow up or death

^c^*N* = 3 of 4 *SUMO4*:c.2T>C carriers were assessed by ALSFRS-R

^d^*N* = 183 of 218 *SUMO4*:c.2T>C non-carriers were assessed by ALSFRS-R

^e^Environmental stress factors comprised (i) head trauma and (ii) tumors with and without treatment by radiation and/or chemotherapy

^f^Listed in Table 3

^*^Significant difference at *p* ≤ 0.05 (given in bold print). Comparisons between *SUMO4*:c.2T>C variant carriers and non-carriers were made using the Mann–Whitney *U* test for continuous variables or the two-tailed Fisher’s exact test for dichotomous variables

**Table 3:** Environmental oxidative stress-related factors observed in three of four ALS patients carrying the *SUMO4*:c.2T>C variant, age of stress occurrence and ALS onset

*SUMO4*:c.2T>C carrier	Stress factor	Age at which stress occurred (in years)	Age at ALS onset (in years)
MD022	Rectal cancer treated by radiochemotherapy	60	71
VALS051	Metastatic prostate cancer treated by radiochemotherapy	69	73
TALS004-01	Head trauma	6	29

*ALS* amyotrophic lateral sclerosis

To analyze the consequences of the heterozygous *SUMO4*:c.2T>C initiator codon variant, RNA was extracted from whole blood of patient TALS004-01, who was still alive, and two ALS patients not carrying the *SUMO4* variant. By real-time reverse transcription PCR, *SUMO4* mRNA levels of *SUMO4*:c.2T>C variant carrier TALS004-01 were significantly lower than mean levels of the two non-carriers (Fig. 1b). These results were confirmed on the protein level by Western blot analysis of lysates of skin biopsy-derived fibroblasts of patient TALS004-01, which were also shown to harbor the *SUMO4*:c.2T>C variant (Fig. 1c) and to contain significantly decreased unconjugated SUMO4 protein levels compared to fibroblasts of two sex- and age-matched non-ALS controls without rare *SUMO4* variants (Fig. 1d, e). Similar results were obtained using a pan-SUMO antibody detecting all SUMO isoforms, i.e., SUMO1-4, although not statistically significant (Fig. 1d, e). Taken together, we show diminished *SUMO4* mRNA and protein expression due to the heterozygous *SUMO4*:c.2T>C variant, apparently not fully compensated by an increased expression of other SUMO isoforms.

In three of four *SUMO4* variant carriers, environmental stress factors were identified as potential additional triggers of ALS pathogenesis (Tables 2, 3). Therefore, we investigated whether the cellular response to stress was altered in fibroblasts of *SUMO4* variant carrier TALS004-01. Using immunofluorescence and the SG marker nucleolysin TIAR, SGs were quantified in fibroblasts of patient TALS004-01 and sex- and age-matched non-ALS controls at baseline, upon arsenite-induced oxidative stress, and after recovery (Fig. 1f). The mean number of TIAR-positive SGs per cell was significantly higher in fibroblasts of patient TALS004-01 compared to control fibroblasts in all three conditions, i.e., baseline, arsenite treatment, and recovery (Fig. 1g). These data demonstrate persistent SGs, increased SG accumulation upon stress exposure, and reduced SG dynamics after stress challenge in fibroblasts of an ALS patient carrying the *SUMO4*:c.2T>C variant.

As SUMOylation of the ALS-associated protein VCP by SUMO1 was previously reported [5], we analyzed whether SUMO4 is also conjugated to VCP. Our analysis of VCP-immunoprecipitates from fibroblast lysates demonstrates for the first time that VCP is also SUMOylated by SUMO4 (Fig. 1h). Consistent with the lower unconjugated SUMO4 levels in fibroblasts of *SUMO4* variant carrier TALS004-01, SUMOylation of VCP by SUMO4 was significantly reduced in these cells compared to sex- and age-matched non-ALS control fibroblasts (Fig. 1h, i).

To further establish whether *SUMO4* variants may play a role in a neurodegenerative disease such as ALS, *SUMO4* mRNA expression was quantified in different human tissues. By real-time PCR*, *the highest human *SUMO4* mRNA levels were observed in brain, followed by placenta and skeletal muscle (Fig. 1j).

## Discussion

Here, we demonstrate that a heterozygous initiator codon variant in the *SUMO4* gene is significantly more frequent in 222 European ALS patients than in two control cohorts from Europe. *SUMO4* variants have previously been associated with human disease in that the *SUMO4* Met55Val variant was implicated in the susceptibility to type I diabetes mellitus [15, 16]. *SUMO4* encodes one of four human SUMO isoforms that are attached to more than a thousand proteins in human cells and are essential for the regulation of several cellular processes such as transcription and mRNA processing [6, 8]. As may be expected from a disrupted initiation codon shown to diminish gene expression in the case of an ATG to ATA conversion at the *Fgf5* locus [17], the consequences of the heterozygous *SUMO4*:c.2T>C variant were reduced *SUMO4* mRNA and protein levels. This variant effect does not seem to be fully compensated by an increased expression of other SUMO isoforms consistent with *SUMO4* haploinsufficiency as a contributor to ALS pathogenesis. In addition, we identified that the stress factors head trauma or cancer treated by radiochemotherapy, which are discussed as potential additional triggers of ALS pathogenesis via an increased generation of free radicals [13], occurred in carriers of the *SUMO4* initiator codon variant prior to ALS onset. Therefore, the combination of this genetic alteration with environmental factors contributing to oxidative stress may cause ALS in these patients.

SGs that form in response to various cellular stress conditions, including heat or oxidative stress, are increasingly associated with neurodegenerative diseases, particularly ALS [1]. Interestingly, the SUMO system may control both the assembly and dissolution of SGs [6]. In line with this notion, our data link *SUMO4* haploinsufficiency in an ALS patient to persistent SGs, excessive SG accumulation upon stress, and reduced dynamics after stress challenge. Similarly, altered SG dynamics were shown to be a consequence of ALS-associated mutations in another gene, e.g., in *FUS* [3, 4]. Moreover, SG clearance by VCP was reduced by ALS-associated *VCP* mutations [7], which also inhibited the SUMOylation of VCP by SUMO1 [5]. Here, SUMOylation of VCP by SUMO4 was reported and found to be diminished in ALS patient fibroblasts harboring the *SUMO4* initiator codon variant, suggesting that this variant may similarly affect SUMOylation of VCP with relevance for SG disassembly, as do ALS-associated *VCP* mutations.

*SUMO4* expression was previously detected in human kidney [15], immune-related tissues [16], and placenta [18]. The fact that the brain showed the highest *SUMO4* mRNA levels of all human tissues analyzed in our study establishes that *SUMO4* variants may impact the brain, and that *SUMO4* haploinsufficiency may cause neurological symptoms.

In summary, we identified the rare initiator codon variant *SUMO4*:c.2T>C at a significantly higher frequency in a European cohort of ALS patients than in controls. Our findings are based on a limited number of ALS patients and require confirmation in larger cohorts. Here, we introduce the *SUMO4*:c.2T>C variant leading to *SUMO4* haploinsufficiency as a novel potential genetic risk factor for ALS that possibly acts in conjunction with the oxidative stress-related factors trauma and cancer (plus radiochemotherapy), and provide evidence of an impact on SG dynamics and SUMOylation of VCP as similarly shown for mutations in the ALS-linked genes *FUS* and *VCP* [3–5, 7]. Our data corroborate the notion that the SUMO system may protect from stress insults potentially reducing ALS risk.

## Data Availability

Anonymized data from the present study can be made available upon reasonable request after contact with the corresponding authors.
